# Development and Validation of an Early Scoring System for Prediction of Disease Severity in COVID-19 Using Complete Blood Count Parameters

**DOI:** 10.1109/ACCESS.2021.3105321

**Published:** 2021-08-16

**Authors:** Tawsifur Rahman, Amith Khandakar, Md Enamul Hoque, Nabil Ibtehaz, Saad Bin Kashem, Reehum Masud, Lutfunnahar Shampa, Mohammad Mehedi Hasan, Mohammad Tariqul Islam, Somaya Al-Maadeed, Susu M. Zughaier, Saif Badran, Suhail A. R. Doi, Muhammad E. H. Chowdhury

**Affiliations:** Department of Electrical EngineeringQatar University 61780 Doha Qatar; Department of Biomedical EngineeringMilitary Institute of Science and Technology 118868 Dhaka 1216 Bangladesh; Department of Computer Science and EngineeringBangladesh University of Engineering and Technology 61750 Dhaka 1205 Bangladesh; Faculty of Robotics and Advanced ComputingQatar Armed Forces-Academic Bridge Program, Qatar Foundation Doha Qatar; COVID Isolation UnitUnited Hospitals, Ltd. Dhaka 1212 Bangladesh; Department of Obstetrics and GynecologyDhaka Medical College Hospital (COVID UNIT) Dhaka 1000 Bangladesh; Dhaka North City Corporation COVID Hospital Dhaka 1208 Bangladesh; Department of Electrical, Electronics and Systems EngineeringUniversiti Kebangsaan Malaysia 61775 Bangi Selangor 43600 Malaysia; Department of Computer Science and EngineeringQatar University 61780 Doha Qatar; Department of Basic Medical SciencesCollege of MedicineQU Health, Qatar University 61780 Doha Qatar; Department of Plastic SurgeryHamad Medical Corporation Doha Qatar; Department of Population MedicineCollege of MedicineQU Health, Qatar University 61780 Doha Qatar

**Keywords:** Complete blood count, prognostic model, machine learning, early prediction of mortality risk, COVID-19

## Abstract

The coronavirus disease 2019 (COVID-19) after outbreaking in Wuhan increasingly spread throughout the world. Fast, reliable, and easily accessible clinical assessment of the severity of the disease can help in allocating and prioritizing resources to reduce mortality. The objective of the study was to develop and validate an early scoring tool to stratify the risk of death using readily available complete blood count (CBC) biomarkers. A retrospective study was conducted on twenty-three CBC blood biomarkers for predicting disease mortality for 375 COVID-19 patients admitted to Tongji Hospital, China from January 10 to February 18, 2020. Machine learning based key biomarkers among the CBC parameters as the mortality predictors were identified. A multivariate logistic regression-based nomogram and a scoring system was developed to categorize the patients in three risk groups (low, moderate, and high) for predicting the mortality risk among COVID-19 patients. Lymphocyte count, neutrophils count, age, white blood cell count, monocytes (%), platelet count, red blood cell distribution width parameters collected at hospital admission were selected as important biomarkers for death prediction using random forest feature selection technique. A CBC score was devised for calculating the death probability of the patients and was used to categorize the patients into three sub-risk groups: low (<=5%), moderate (>5% and <=50%), and high (>50%), respectively. The area under the curve (AUC) of the model for the development and internal validation cohort were 0.961 and 0.88, respectively. The proposed model was further validated with an external cohort of 103 patients of Dhaka Medical College, Bangladesh, which exhibits in an AUC of 0.963. The proposed CBC parameter-based prognostic model and the associated web-application, can help the medical doctors to improve the management by early prediction of mortality risk of the COVID-19 patients in the low-resource countries.

## I. Introduction

COVID-19 disease recorded in Wuhan, China, in December 2019 has quickly spread throughout the world while some parts of the world are even suffering from the second and third waves of the pandemic. As of July 12, 2021, the worldwide confirmed cases are 187 millions in more than 206 countries with 4.03 millions deaths caused by COVID-19 [1]. COVID-19 was declared a pandemic by the World Health Organization (WHO) on March 11, 2020 [2]. The coronavirus mostly affects the lungs of the patients and leads to pneumonia [3]. The majority of patients were mildly affected by the disease with common respiratory symptoms [4]. Fever and cough are the most common clinical symptoms. There are around 20 % of the cases, where the radiographic chest images did not show any abnormalities in the initial stages of the COVID-19 infected patients [5]. Serious cases should meet one or more of the following procedures, according to the sixth edition of the Novel Coronavirus Pneumonia Diagnosis and Treatment Plan: 1) shortness of breath (30 breaths per minute), 2) oxygen saturation (93 percent at rest), or 3) arterial partial pressure of oxygen/fraction of inspired oxygen (300 mm Hg) [6]. Roughly 10–15% patients associated with severe outcomes showed extreme conditions such as severe pneumonia, Acute respiratory distress syndrome (ARDS), or multiple organ failure, before or during hospitalization [7]–[9]. A large cohort study from 2449 patients showed that large hospitalization (20–31 %) and intensive care unit (ICU) admission rates (4.9–11.5%) have overwhelmed the healthcare system [8]. This can be prevented by prioritizing hospital care for patients who are at high risk of deterioration and death while treating the low-risk patients in ambulatory settings or at home-based self-quarantine facilities. As a result, specific predictive methods for predicting the risk of severe COVID-19 infection are urgently needed [7].

Several studies have shown that biomarkers can assist in the classification of COVID-19 patients with an increased risk of serious disease and mortality by providing a vital information about their health status. Clinical machine-learning-based nomograms have been developed and proposed by several groups [10], [11], which allows parameter-based risk estimation, thus easing the decision-making process for the management. Zheng *et al.* [12] showed from 141 patients of Zhejiang, China that the white blood cell count, neutrophil count, and platelet counts were at the normal range for 87.9%, 85.1%, and 88.7% of the patients, respectively. Among the severe patients, 82.8 percent had lymphopenia, which pronounces with disease progression. A scoring system called NLP based on neutrophil, lymphocyte, and platelet counts has been shown to be useful in patient stratification. This model was developed on a small dataset and was not validated on any external dataset. Al Youha *et al.* [13] proposed the Kuwaiti Progression Indicator (KPI) Score as a prognostic model for predicting COVID-19 severity progression. Unlike other self-reported symptoms and arbitrary parameter-driven scoring schemes, the KPI model was based on quantifiable laboratory readings. The KPI score categorizes patients as low risk if their score is below −7 and high risk if their score is above 16, but the authors consider the risk of advancement in the intermediate category (patients with scores between −6 and 15) to be unknown. Many prognostic systems, however, fall into this intermediate category. Weng *et al.* [14] used 301 adult patients to build an early prediction score called ANDC to predict mortality risk for COVID-19 patients. Age, neutrophil-to-lymphocyte ratio (NLR), D-dimer, and C-reactive protein reported during admission were identified as mortality predictors for COVID-19 patients using least absolute shrinkage and selection operator (LASSO) regression [14]. A nomogram with an integrated score, ANDC was proposed to ascertain death probability, which demonstrated a good association between the true and predicted output. Two cut-off values of the ANDC score were used to divide COVID-19 patients into three risk categories: low, moderate, and high. In the low-risk, moderate-risk, and high-risk groups, the death likelihood was 5%, 5% to 50%, and more than 50%, respectively. Ramachandran *et al.* [15] showed that elevated Red Blood Cell Distribution Width (RDW) in hospitalized COVID-19 patients is associated with a substantially increased risk of mortality and septic shock. However, other blood count parameters, which were not mentioned in this article, should be investigated in relation to RDW. Based on 372 COVID-19 patients from China, Gong *et al.* [16] showed that one demographic and six serological markers (serum lactate dehydrogenase, C-reactive protein, the coefficient of variation of red blood cell distribution width (RDW), blood urea nitrogen, albumin, and direct bilirubin) were linked to extreme COVID-19. However, the performance of the reported models degraded on the validation cohort. Both elevated RDW at admission and diagnosis were found related to an increased mortality risk based on 1,198 adult patients diagnosed with COVID-19 from four hospitals between March 4, 2020, and April 28, 2020 [17]. Jianfeng *et al.* [18] proposed a prognostic model using lactate dehydrogenase, lymphocyte count, age, and oxygen saturation (SpO2) as primary predictors of COVID-19-related death based on a cohort of 444 patients. Internal and external validation showed strong discrimination, with C-statistics of 0.89 and 0.98, respectively. However, external validation revealed over- and under-prediction for low-risk and high-risk patients, respectively, even though the model was promising for internal validation. Yan *et al.* [19] used a machine learning method to identify three biomarkers (lactic dehydrogenase (LDH), lymphocytes, and high-sensitivity C-reactive protein (hs-CRP)) and used them to predict individual patients’ mortality 10 days ahead with over 90% precision. High levels of LDH, in particular, have been shown to be important in distinguishing the majority of patients, who need immediate medical attention. However, no scoring system is introduced in this study that can aid clinicians in quantitatively stratifying patients at risk.

Using a cohort of 1,590 patients from 575 medical centers, Liang *et al.* [20] proposed a deep learning model to develop an online calculator for patient triage at admission by identifying the severity of illness. This will ensure that the patients at the highest risk will receive adequate treatment as soon as possible and thereby healthcare resource utilization will be maximized. This model is a very useful technique for patient stratification however, it depends on demography, radiography, and other clinical criteria as well as comorbidity data to make a decision, which is not always accessible to the low-resources countries. Wang *et al.* [21] found that the neutrophils to lymphocytes ratio (NLR) and Red Cell Distribution Width Standard Deviation (RDW-SD) combined parameter is the best hematology index for predicting the severity of COVID-19 patients. However, only 45 COVID-19 patients were included in the study. Huang *et al. [22]* used nine independent risk factors at admission to the hospital to quantify the risk score and stratify the patients into various risk groups in a retrospective, multicenter analysis on 336 confirmed COVID-19 patients and 139 control patients. This research did not use any external validation. The independent relationship between the baseline level of four indicators (NLR, LDH, D-dimer, and Computer tomography (CT) score) on admission and the severity of COVID-19 was assessed using logistic regression technique. The presence of high levels of NLR and LDH in serum could help in the early detection of COVID-19 patients who are at high risk. It was shown that the usage of LDH and NLR together increased the detection sensitivity [23]. This model, however, is based on a CT image-based ranking, which is not available for all patients. In a limited number (84) of hospitalized patients with COVID-19 pneumonia, Liu *et al.* [24] suggested combining the NLR and CRP to predict 7-day disease severity. A retrospective cohort of 80 COVID-19 patients treated at Beijing You’an Hospital was analyzed to identify risk factors for serious and even fatal pneumonia and establish a scoring system for prediction, which was later validated in a group of 22 COVID-19 patients [25]. Age, diabetes, coronary heart disease (CHD), percentage of lymphocytes (%LYM), procalcitonin (PCT), serum urea, CRP, and D-dimer were found to be correlated with mortality by LASSO binary logistic regression in a chort of 2,529 COVID-19 patients. The researchers then used multivariable analysis to determine that age, CHD, %LYM, PCT, and D-dimer were independently posing risk for mortality. A COVID-19 scoring system (CSS) was developed based on the above variables to classify patients into low-risk and high-risk categories with discrimination of AUC = 0.919 and calibration of P = 0.64 [26]. Another study on 82 COVID-19 patients found that respiratory, cardiac, hemorrhage, hepatic, and renal damage were responsible for 100%, 89%, 80.5%, 78%, and 31.7% of deaths, respectively. The majority of the patients had elevated CRP (100%) and D-dimer (97.1%) [25]. D-dimer is shown as a prognostic factor which has also been shown to substantially increase the chances of death if it is greater than $1~\mu \text{g}$ mL $^{-1}$ at the time of admission [27], [28]. While several predictive prognostic models for early detection of individuals at high risk of COVID-19 mortality have been proposed, there is still a significant gap in the prediction model based on complete blood count (CBC) parameters based on detailed interpretable machine learning based models and quantitative scoring framework. Measurement of multiple biomarkers for a large number of patients is difficult in different countries and healthcare facilities. This is a critical problem for low-resource countries (LRCs), thus it was interesting to see how well a model based on CBC parameters could stratify the risk-factor of COVID-19 patients compared to a standard model based on all of the parameters recorded in the literature. No previous studies have evaluated the important bio-markers among CBC parameters as early warning models for predicting the risk of severe COVID-19, to the best of our knowledge.

Important CBC biomarkers were identified using machine learning algorithms in order to develop an early prediction based scoring technique, which can stratify the patients into risk groups. This can assist in better patient care based on easily accessible CBC biomarkers. The top-ranked CBC features with the best classification performance were used to construct a multivariable logistic regression-based nomogram to predict the risk of death. The results of this study include a quick, easy-to-use, and accurate algorithm for predicting high-risk individuals and can help in the efficient utilization of healthcare resources.

## II. Methodology

**FIGURE 1.**
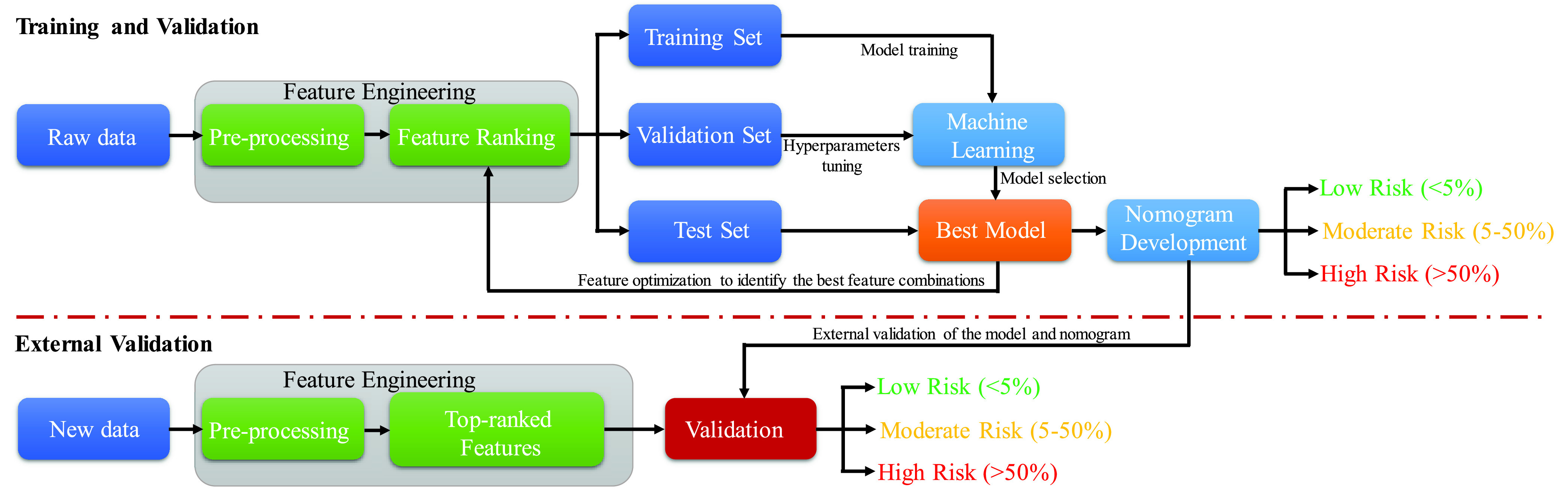
Schematic diagram of the experimental frame work.

The authors have used a publicly available clinical dataset from China to develop the machine learning model and scoring techniques in this study, details of which is provided later. Moreover, the authors have collected a dataset in collaboration with medical doctors from different COVID-19 care centers in Bangladesh for external validation. Firstly, the Chinese raw dataset was pre-processed before experimenting with various popular feature ranking techniques and machine learning models. The pre-processing includes filling the missing data using data imputation techniques and then normalize the imputed data for feature ranking and classification. The best performing combination of the features (with the help of popular feature ranking techniques) and machine learning classifiers were investigated. The best performing logistic regression classifier was used to develop a multi-variate nomogram based scoring technique to detect the risk of mortality due to COVID-19. The developed nomogram is then further validated with the completely unseen external dataset of Bangladeshi population to confirm its robust performance. The details of the complete methodology is shown in Figure 1.

### A. Study Design and Patients

Firstly, this retrospective study was performed in the COVID-19 healthcare center for confirmed patients in Wuhan at the center of the outbreak in China. Blood samples and Medical records were collected from 375 patients from 10 January 2020 to 18 February 2020. Epidemiological, demographic, clinical, laboratory, and mortality outcomes were recorded from electronic health records. This dataset of 375 COVID-19 patients were made public by Yan *et al.* [19] and the study was approved by the Tongji Hospital Ethics Committee.

Secondly, a retrospective study was performed between 12 April and 31 August 2020 at Dhaka Medical College Hospital, Bangladesh which is approved by the Hospital Ethical Committee. Clinical parameters with hospital admission, discharge/death outcomes were collected for 103 patients (Survived-61 (59.22%), Death-42 (40.78%)) and these data were used for external validation. The Bangladeshi dataset is made publicly available by the authors and can be found in [29].

Exclusion criteria were individuals who were not hospitalized or treated, below 18 years old, pregnant, had less than 20% data, and on breastfeeding. Among 375 patients, different patients have showed different symptoms: fever (49.9%), cough (13.9%), fatigue (3.7%), dyspnea (2.1%), chest distress (1.9%) and muscular soreness (0.5%).

### B. Statistical Characteristics

Statistical analysis of the patients’ demographic, clinical, and outcome data was carried out by Stata/MP 13.0 software. Gender, age, and twenty-three complete blood count (CBC) parameters were identified from the Chinese database which has seventy-six bio-markers. Gender differences in data were described using percentage while other variables were characterized using missing data, the mean and standard deviation for survival and death outcomes. Univariate analysis was conducted on gender while Wilcoxon’s ranked tests were done on the rest of the variables. P-value was calculated on a 95% statistical significance threshold and therefore P-value should be less than 0.05 to be considered as significant. Table 1(A) summarizes 25 parameters (age, gender, and CBC markers) and their statistical characteristics. Table 1(B) summarizes the details of the important features of the Dhaka Medical College dataset that was used to validate the developed model in this work.

**TABLE 1:** (A): Statistical Characteristic Analysis of COVID-19 Patients in Survival and Death Groups Using Chinese Data. (B): Statistical Characteristic Analysis of COVID-19 Patients in Survival and Death Groups Using Bangladeshi Data

Item	Survived	Death	Total	Method	Statistic	P value
Gender • Male (%) • Female (%)	98(49%) 103(51%)	126(72%) 48(28%)	224(60%) 151(40%)	Chi-square test	$\chi ^{2}=21.70$	<.05
Age (years) • N(missing) • Mean ± SD	201(0) 50.2±15	174(0) 68.8±11.8	375(0) 58.8±16.5	Rank-sum test	Z=−11	<.05
Hemoglobin (g/dL)N • (missing) • Mean ± SD	194(7) 12.4±2.64	162(12) 12.6±2.33	356(19) 12.5±2.5	Rank-sum test	Z=−0.67	0.502
Red blood cell count ( $\times 10^{6} /\mu \text{L}$) • N(missing) • Mean ± SD	194(7) 4.4±1.8	163(11) 5.4±8.51	357(18) 4.8±5.91	Rank-sum test	Z=0.118	0.907
Mean corpuscular volume (fL) • N(missing) • Mean ± SD	194(7) 88.8±5.41	162(12) 89.7±6.73	356(19) 89.2±6.06	Rank-sum test	Z=−1.84	0.067
Mean corpuscular hemoglobin (pg) • N(missing) • Mean ± SD	194(7) 30.5±2.44	162(12) 31.1±3.15	356(19) 30.8±2.8	Rank-sum test	Z=−2.77	<.05
Mean corpuscular hemoglobin concentration • N(missing) • Mean ± SD	194(7) 34.3±1.39	162(12) 34.6±1.87	356(19) 34.5±1.63	Rank-sum test	Z=−2.27	0.023
Red blood cell distribution width (%) • N(missing) • Mean ± SD	191(10) 12.37±1.01	159(15) 13.4±1.94	350(25) 12.8±1.58	Rank-sum test	Z=−6.89	<.05
White blood cell count ( $\times 10^{3}/\mu \text{L}$) • N(missing) • Mean ± SD	194(7) 5.6±6.35	163(11) 12±11.6	357(18) 8±9.63	Rank-sum test	Z=−10.65	<.05
Neutrophils count ( $\times 10^{ {3}} /\mu \text{L}$) • N(missing) • Mean ± SD	194(7) 3.5±2.18	162(12) 9.8±5.66	356(19) 6.4±5.18	Rank-sum test	Z=−11.74	<.05
Neutrophils (%) • N(missing) • Mean ± SD	194(7) 65.7±13.8	162(12) 87±9.86	356(19) 75.4±16.1	Rank-sum test	Z=−12.88	<.05
Lymphocyte count ( $\times 10^{ {3}}/\mu \text{L}$) • N(missing) • Mean ± SD	194(7) 1.4±3.72	162(12) 0.61±0.336	356(19) 1±2.78	Rank-sum test	Z=3.39	<.05
Lymphocyte (%) • N(missing) • Mean ± SD	194(7) 24.8±11.4	162(12) 7.6±6.22	356(19) 17±12.7	Rank-sum test	Z=11.97	<.05
Monocyte count ( $\times 10^{ {3}} /\mu \text{L}$) • N(missing) • Mean ± SD	194(7) 0.6±2.42	162(12) 0.45±0.33	356(19) 0.5±1.8	Rank-sum test	Z=−0.49	0.622
Monocyte (%) • N(missing) • Mean ± SD	194(7) 8.4±3.15	162(12) 5.1±4.31	356(19) 6.9±4.08	Rank-sum test	Z=8.42	<.05
Eosinophil count ( $\times 10^{ {3}} /\mu \text{L}$) • N(missing) • Mean ± SD	194(7) 0.035±0.05	162(12) 0.012±.041	356(19) 0.025±0.05	Rank-sum test	Z=−5.66	<.05
Eosinophil (%) • N(missing) • Mean ± SD	194(7) 0.7±.941	162(12) 0.11±0.38	356(19) 0.44±.79	Rank-sum test	Z=6.63	<.05
Basophil count ( $\times 10^{ {3}} /\mu \text{L}$) • N(missing) • Mean ± SD	194(7) 0.011±0.012	152(12) 0.017±0.016	356(19) 0.014±0.015	Rank-sum test	Z=−3.04	<.05
Basophil (%) • N(missing) • Mean ± SD	194(7) 0.22±0.23	162(12) 0.15±0.18	356(19) 0.19±0.21	Rank-sum test	Z=11.98	<.05
Platelet count • N(missing) • Mean ± SD	194(7) 213±82.8	162(12) 161±87.4	356(19) 189±88.7	Rank-sum test	Z=5.40	<.05
PLT distribution width (%) • N(missing) • Mean ± SD	193(8) 12.3±2.02	153(21) 13.6±2.82	346(29) 12.9±2.49	Rank-sum test	Z=−5.41	<.05
Platelet large cell ratio • N(missing) • Mean ± SD	193(8) 29.6±7.12	153(21) 33.8±8.03	346(29) 31.4±7.82	Rank-sum test	Z=−5.81	<.05
ESR • N(missing) • Mean ± SD	158(43) 30.2±21.5	130(44) 39±26.7	288(87) 34±24.3	Rank-sum test	Z=−2.392	<.05
Outcome (%)	201(54%)	174(46%)	375			
Gender • Male (%) • Female (%)	30 (49%) 31 (51%)	25 (59.5%) 17 (40.5%)	55(53.4%) 48(46.6%)	Chi-square test	$\chi ^{2}=18.65$	<.05
Age (years) • N(missing) • Mean ± SD	61(0) 39.9±12.92	42 (0) 57.9±13.3	103 (0) 47.3±15.7	Rank-sum test	Z=−6.75	<.05
Red blood cell distribution width (%) • N(missing) • Mean ± SD	61(0) 12.81±0.8	42 (0) 13.81±1.67	103 (0) 13.37±1.43	Rank-sum test	Z=8.6	<.05
White blood cell count ( $\times 10^{ {3}} /\mu \text{L}$) • N(missing) • Mean ± SD	61(0) 12.5±11.01	42 (0) 11.5±7.6	103 (0) 12.1±9.4	Rank-sum test	Z=7.34	<.05
Neutrophils count ( $\times 10^{ {3}} /\mu \text{L}$) • N(missing) • Mean ± SD	61(0) 7.1±2.6	42 (0) 9.7±5.4	103 (0) 8.2±5.1	Rank-sum test	Z=11.6	<.05
Lymphocyte count ( $\times 10^{ {3}} /\mu \text{L}$) • N(missing) • Mean ± SD	61(0) 3.4±1.4	42 (0) 3.9±3.4	103 (0) 3.5±2.9	Rank-sum test	Z=−5.9	<.05
Monocytes (%) • N(missing) • Mean ± SD	61(0) 3.55±1.2	42 (0) 4.5±2.8	103 (0) 3.94±2.1	Rank-sum test	Z=−9.45	<.05
Platelet count ( $\times 10^{ {3}} /\mu \text{L}$) • N(missing) • Mean ± SD	61(0) 225±35.8	42 (0) 203±77.4	103 (0) 216.3±57.1	Rank-sum test	Z=11.23	<.05
Outcome (%)	61 (59.22%)	42 (40.78%)	103			

### C. Data Pre-Processing

#### 1) Data Imputation and Normalization

Each patient has multiple blood samples however, some patients have some parameters missing while others have different parameters missing. The patient data at admission was used to identify the key predictors of the disease severity. Missing data can be dealt with differently in different types and sizes of data. In the simplest technique, patients with incomplete parameters could be removed from the study if the number of subjects is very large in the study however, it can lead to loss of very useful information of the data [30]. It is a good practice to identify and replace missing data i.e., carry out data imputation prior to modeling for the prediction task. A popular approach to missing data imputation is to use a machine learning model to predict the missing values. This requires a model to be created for each input variable that has missing values. There are several models popularly used for this purpose, such as k-nearest neighbor (KNN), random forest, multiple imputation using chained equations (MICE) data imputation technique, etc. KNN imputation technique was proven to be generally effective for clinical data imputation [31]. Therefore, the KNN technique was used in this study. The hyper-parameters of the KNN algorithm is the distance measure (e.g. Euclidean distance) and the number of contributing neighbors for each prediction. KNN parameters were set to: number of neighbors = 5, weights = ‘uniform’, and distance = Euclidean.

Several data normalization techniques were used to transform the features to be on a similar scale, which improves the performance and training stability of a model. Commonly used techniques are scaling to a range, clipping, log scaling, and z-score normalization. When the dataset does not contain extreme outliers, the Z-score technique is suitable for normalization, and therefore, the ‘Z-score’ technique was used for data normalization. Since the number of patients with death and survival outcomes were not equal or the dataset was imbalaced, therefore, a very popular clinical data augmentation technique called Synthetic Minority Over-sampling Technique (SMOTE) was used to make the dataset balance.

#### 2) Top-Ranked Features Identification

The feature selection technique automatically selects those features which are the most contributing features for predicting the output. This reduces overfitting, improves accuracy, and reduces training time. Several different feature selection techniques are used in the literature, such as univariate selection, recursive feature elimination (RFE), principal component analysis (PCA), bagged decision trees like random forest and extra trees, and boosted trees like Extreme Gradient Boosting (XGBoost), etc. In this study, authors investigated random forest, extra tree and XGBoost techniques. However, random forest provides higher accuracy in selecting top-10 features in the mortality prediction among the 25 features including age, gender, and CBC parameters. As per literature this technique is better suited for datasets with many predictor variables [32].

### D. Selection of Classification Mode

In this study, several supervised machine learning (ML) classification models such as linear discriminant analysis [33], random forest [34], support vector machine (SVM) [35], XGBoost [36], logistic regression classifiers [37] and Multilayer perceptron (MLP) [38] are compared for classification. Linear discriminant analysis (LDA) finds the probability of an input belonging to the various classes and predicts based on the highest probability. SVM is a very popular ML algorithm for different applications for non-linear classification using high-dimensional feature spaces. XGBoost is a supervised ML algorithm that can be used for the training data with multiple features. Logistic regression is a commonly used medical statistics-based supervised ML model, dedicated to classification tasks. The logistic function is a sigmoid function that contracts the real continuous values into a probability of [0, 1] [37]. A multilayer perceptron (MLP) is a type of feedforward artificial neural network (ANN), which is made up of at least three layers of nodes: an input layer, a hidden layer and an output layer. MLP utilizes a supervised learning technique called backpropagation for training [38].

Different classification models were compared using the top-10 ranked features from the testing data to calculate the performance matrices in classifying death and survival cases. The best performing classifier among the aforementioned classifiers was evaluated for different combinations of features as input to the model by calculating the receiver operating characteristic (ROC) - area under the curve (AUC) and performance metrics such as Precision, Sensitivity, Specificity, Accuracy, and F1-Score. Since the model development dataset was made balance using SMOTE technique [39], the threshold for the ROC calculation was 0.5 [40]. Different classification algorithms and different features’ combination of the best performing algorithm were validated using 5-fold cross-validation where training and testing were done on 80% and 20% data, respectively, and this process was repeated 5-times to test the entire dataset. Since some CBC parameters are present in count and percentage forms, top-ranked 10 features from 25 feature sets were identified and investigated with count parameters and with percentage parameters. Weighted average within 95% confidence interval was calculated for sensitivity, specificity, precision, F1-score, and overall accuracy from the confusion matrix that accumulates all test (unseen) fold results of the 5-fold cross-validation.

\begin{align*} Sensitivity=&\frac {TP}{TP+FN}\tag{1}\\ Specificity=&\frac {TN}{TN+FP}\tag{2}\\ Precision=&\frac {TP}{TP+FP}\tag{3}\\ F1\_{}score=&2\frac {Precision\times Sensitivity}{Precision+Sensitivity}\tag{4}\\ Accuracy=&\frac {TP+TN}{TP+TN+FP+FN}\tag{5}\end{align*}

Here, the correct mortality prediction of dead patients is True Positive(TP), and the correct mortality prediction of survived patients is True Negative (TN). The incorrect mortality prediction of dead patients as survived is False Negative (FN) and the incorrect mortality of survived patients as dead is False Positive (FP).

### E. Logistic Regression-Based Nomogram

Nomogram is a two-dimensional graphical tool that consists of several lines scaled and arranged in such a way to be used to predict the outcome probability. This is an important component of modern medical decision-making. In this work, a multivariate logistic regression analysis based nomogram technique was used, which was originally developed by Alexander Zlotnik in Stata/MPv13.0 [41]. The parameters were drawn as a numerated horizontal axis scale and the values for the patient are put on the numerated scale. A vertical line was drawn down from the different horizontal lines to a score axis. All the scores on the score axis were added to make a total score and this was linked to a death probability. It can be noted that a higher score corresponds to a higher death probability.

Logistic regression is a statistical model that in its basic form uses a logistic function to model a binary dependent variable. Logistic regression uses input values (x) that are combined linearly using weights or coefficient values to predict an output value (y). In a logistic regression model, the outcome variable is modeled to binary values (0 or 1) and the odds are defined by the ratio of the probability (P) of happening an event to the probability of not happening (1-P). Therefore, the probability can vary between 0 and 1 but the odds vary between 0 to infinity. The natural logarithm of odds is the linear prediction which is a linear combination of binary (e.g., gender) or continuous (e.g., age) predictors. Linear prediction can be used to calculate the death probability, as shown below:

\begin{align*} Linear ~Prediction (LP)=&\ln \left ({odds }\right)=\ln \left ({\frac {P}{1-P} }\right)=b_{0} \\&+\,b_{1}x_{1}+b_{2}x_{2}+\cdots +b_{n}x_{n}\quad \tag{6}\\ P=&\frac {1}{1+e^{-LP}}\tag{7}\end{align*}

Different investigations were carried out to identify the best feature combinations for creating the nomogram. The best feature combination was selected based on the performance matrix and the AUC calculated from the ROC curve. To develop the nomogram and validate its’ performance, the entire Chinese dataset was divided into two subsets: training (70%) and internal validation (30%). Dhaka Medical College hospital patient cohort was used for external validation. Calibration curves were plotted using the internal and external validation sets to compare the model performance of predicting the outcomes compared to the actual outcomes for patients with COVID-19. Decision curve analysis (DCA) was performed to obtain the threshold values of each CBC parameter individually and in combination to evaluate the model performance using Stata/MPv13.0.

### F. Early Warning CBC Score

In the development of the prognostic model, CBC parameters derived from an initial blood sample of the patients at their admission were used. However, these patients have multiple blood samples recorded during their hospital stay, which can be used for longitudinal model evaluation as an early predictor of the patients’ outcome.

The corresponding probability of death for a given CBC score was determined from the model and two cut-off values were identified based on 5% and 50% of death probability and associated CBC score to group the patients into three groups, such as low, moderate, and high-risk groups. The death probability less than 5% is considered to be in the low-risk group, while probability between 5% and 50% is considered moderate risk group and finally the probability above 50% is considered to be in the high-risk group.

## III. Results

### A. Patients’ Characteristics and Outcome

There are two sets of data used in this study: one was 375 patients from Wuhan Hospital, China and the other one was 103 patients from Dhaka Medical College Hospital, Bangladesh. 375 COVID-19 positive hospital admitted patients were used for model development and internal validation, where 46.4% (174) were died and 53.6% (201) were discharged from hospital after recovery. The model was externally validated on 103 COVID-19 positive patients, where 59.2% (61) patients were survived and 40.8 % (42) patients were died. For 375 patients from Wuhan hospital, th minimal, maximal, and median hospital stay of the patients before outcomes (death or discharge) were 0 days, 35 days, and 12 days, respectively. On the external validation set, for 103 patients, the minimum, maximum, and median hospital stay before death or discharge were 5 days, 25 days, and 9 days.

Depending on the patient’ outcome, 375 patient’ data were summarized in Table 1(A).59.7% (224) and 40.3% (151) patients who were male and female, respectively with a mean age of 58.83 ± 16.46 years. 76 demographic and laboratory parameters are available in the development dataset however, only 23 CBC parameters and two demographic parameters were used for this study.

Missing variables in the dataset were imputed using the KNN algorithm. Detailed characteristics of the 25 parameters were listed in Table 1 and it was evident from the chi-square and ranked-sum test that some parameters are statistically insignificant (p > 0.05) while others are statistically significant (p < 0.05) in predicting the death outcomes of the patients. It was found that age, gender, neutrophils (%), lymphocyte (%), eosinophil (%), monocyte (%), platelet count, red blood cell distribution width, white blood cell count, mean platelet volume, basophil (%), platelet large cell ratio, PLT distribution width, eosinophil count, neutrophils count, mean corpuscular hemoglobin, ESR, basophil count, and lymphocyte count had a statistically significant difference between death and survival group while hemoglobin (%), Mean corpuscular volume, red blood cell count, mean corpuscular hemoglobin concentration, and monocyte count are statistically insignificant among the different groups.

Table 1(B) summarizes the data of 103 patients based on their outcomes. Patients were 53.4 % (55) male and 46.6 % (48) female, with a mean age of 47.3 ± 15.7 years. The validation dataset included 15 demographic and laboratory parameters, but only 6 CBC parameters and two demographic parameters were used in this study.

### B. Selection of Classifier and Feature Ranking and Tuning

**FIGURE 2.**
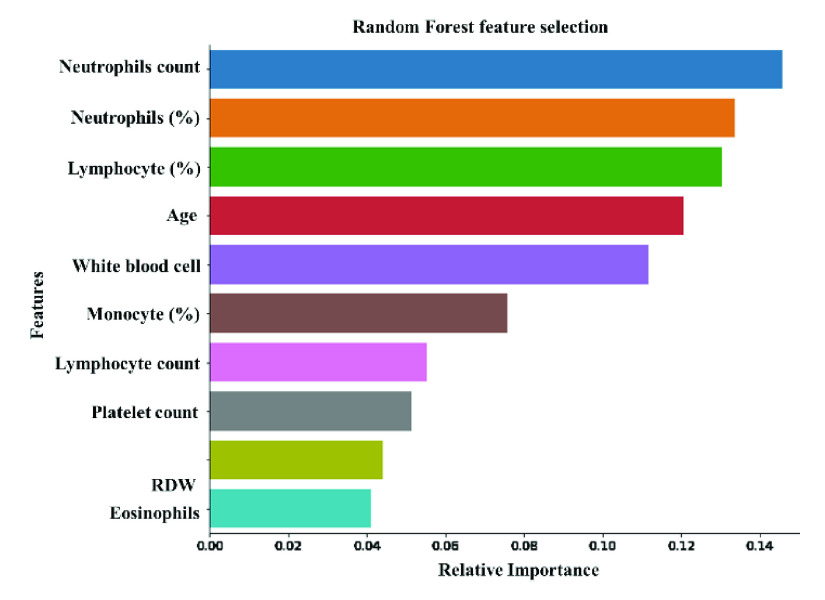
Top-ranked-10 features using random forest feature selection technique.

A random forest feature ranking algorithm was used to identify top-ranked 10 features among the 16-statistically significant features (Figure 2). These top-ranked 10 features were investigated with 5 different classifiers to identify the best performing classification model and results are reported in Table 2. Logistic regression is outperforming other networks in the binary classification problem using the top-ranked 10 features. It provides overall accuracy, and weighted precision, sensitivity, specificity, and F1-score of 88%, 88%, 87%, and 90% respectively. In the rest of the study, therefore, logistic regression was used as the classifier. It was also important to check the most useful variables for the early prediction of death among the top-10 features.

**TABLE 2:** Comparison of Performance Evaluation Parameters of Five Different Algorithms for Classifying Death/Survival Outcomes

	Weighted Average (95% confidence interval)					Confusion Matrix			
	Precision	Sensitivity	f1-score	Specificity	Overall Accuracy	Survived		Death	
						TN	FP	FN	TP
SVM	85±2.10	84±3.18	84±4.13	84±5.08	84	159	42	18	156
Linear Discriminant Analysis	85±2.12	86±3.06	86±2.23	85±4.1	86	174	27	26	148
Logistic Regression	88±3.15	87±4.08	87±4.2	90±3.14	88	180	21	23	151
Random Forest	87±2.64	87±3.42	86±2.7	87±3.2	87	175	26	23	151
XGBoost	86±2.03	85±3.13	86±3.03	86±3.5	86	176	25	27	147

**FIGURE 3.**
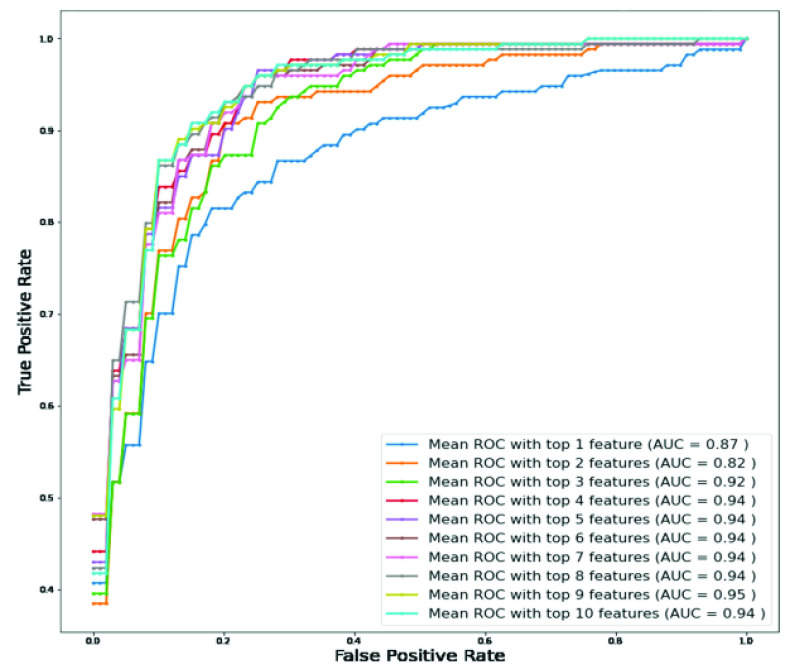
ROC curves for top-10 features using logistic regression classifier (Imputation-KNN, Feature selection–Random forest).

**FIGURE 4.**
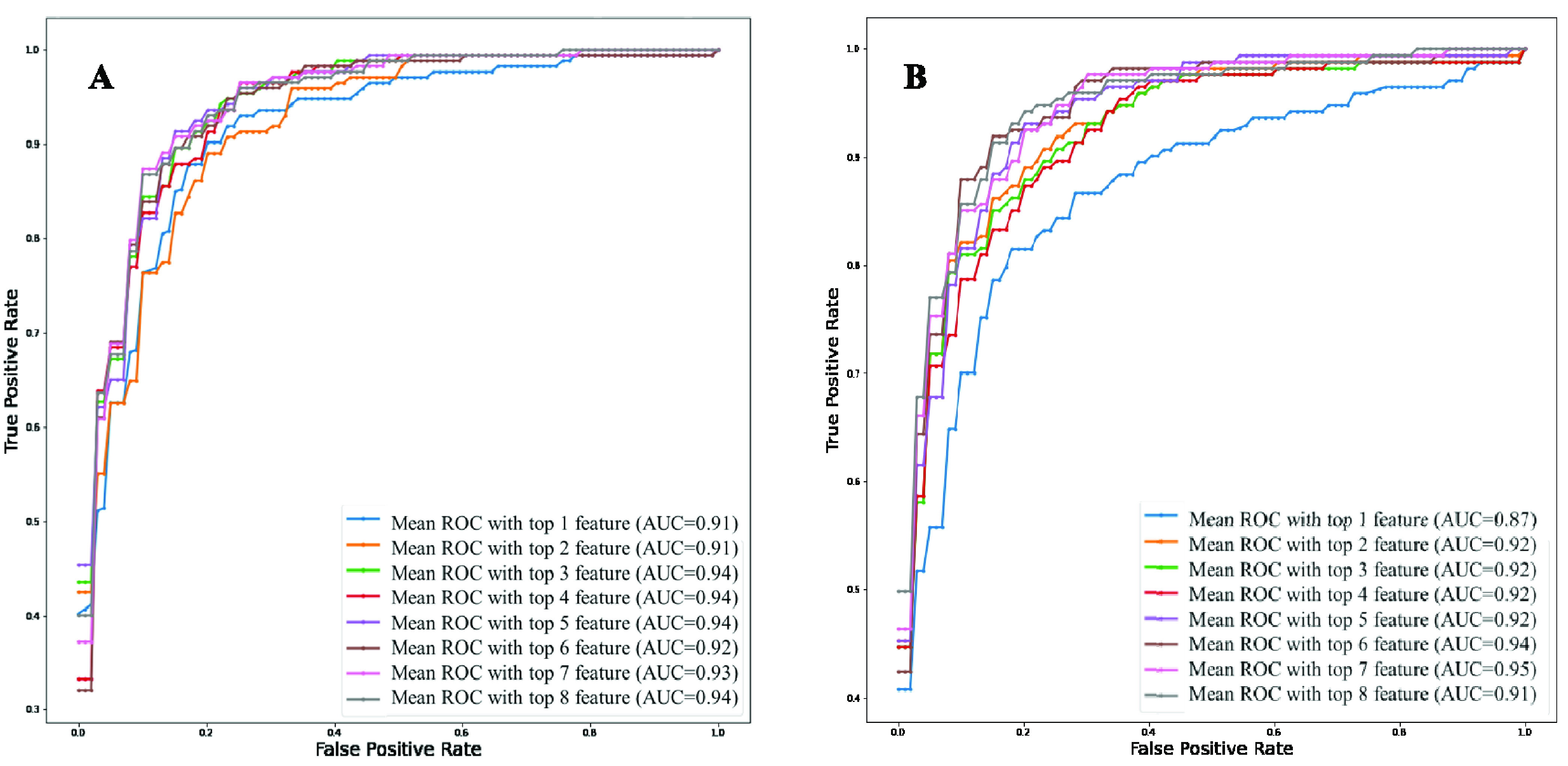
Comparison of the top-ranked 8 features identified using random forest algorithm from data imputed using KNN algorithm A) without neutrophils and lymphocyte counts and B) without neutrophils and lymphocytes percentage.

To determine the association of the independent variables with the outcomes, classification using logistic regression was performed with Top-1 to Top-10 features. Figure 3 clearly shows that the top-ranked 9 features produce the highest value of AUC (0.95). Table 3 shows the overall accuracies and weighted average performance for the other matrices for the different models using Top 1 to 10 features for 5-fold cross-validation using the logistic regression classifier. Top-9 features produce the best performance with AUC = 0.95 and verall accuracy, and weighted precision, sensitivity, specificity, and F1-score of 90%, 90%, 91%, 90%, and 90%, respectively (Table 3). However,both neutrophils and lymphocytes were present in percentage and count in those top-9 features. Therefore, it is necessary to investigate the performance of those features with and without the percentage of neutrophils and lymphocytes. Figure 4(A) shows the ROC curves for the best 8-features considering neutrophils and lymphocytes as a percentage only (excluding neutrophils and lymphocytes as count) while Figure 4(B) shows the ROC curves for the best 8-features considering neutrophils and lymphocytes as count only (without neutrophils and lymphocytes as a percentage).

**TABLE 3:** Overall Accuracy and Weighted Average Performance for Top 1 to 10 Features

	Weighted Average (95% confidence interval)					Confusion Matrix			
	Precision	Sensitivity	f1-score	Specificity	Overall Accuracy	Survived		Death	
						TN	FP	FN	TP
Top 1 feature	86±4.10	78±3.18	78±4.13	90±3.012	82	181	20	49	125
Top 2 features	82±4.06	83±2.16	82±3.08	84±3.03	83.2	168	33	30	144
Top 3 features	83±3.08	83±2.16	82±3.06	84±3.6	83.5	169	32	30	144
Top 4 features	84±3.51	87±2.07	85±3.5	86±3.13	86.13	172	29	23	151
Top 5 features	84±4.11	87±2.06	85±3.72	85±2.11	86	171	30	23	151
Top 6 features	84±4.08	87±3.04	85±3.78	85±3.08	86.13	171	30	22	152
Top 7 features	88±3.10	84±4.08	86±4.02	90±3.09	87	180	21	28	146
Top 8 features	88±3.12	87±3.07	87±3.45	90±3.11	88.2	180	21	23	151
Top 9 features	90±4.14	91±3.10	90±3.5	90.4±3.7	90.4	183	18	18	156
Top 10 features	88±3.15	87±4.08	87±4.2	90±3.14	88	180	21	23	151

**FIGURE 5.**
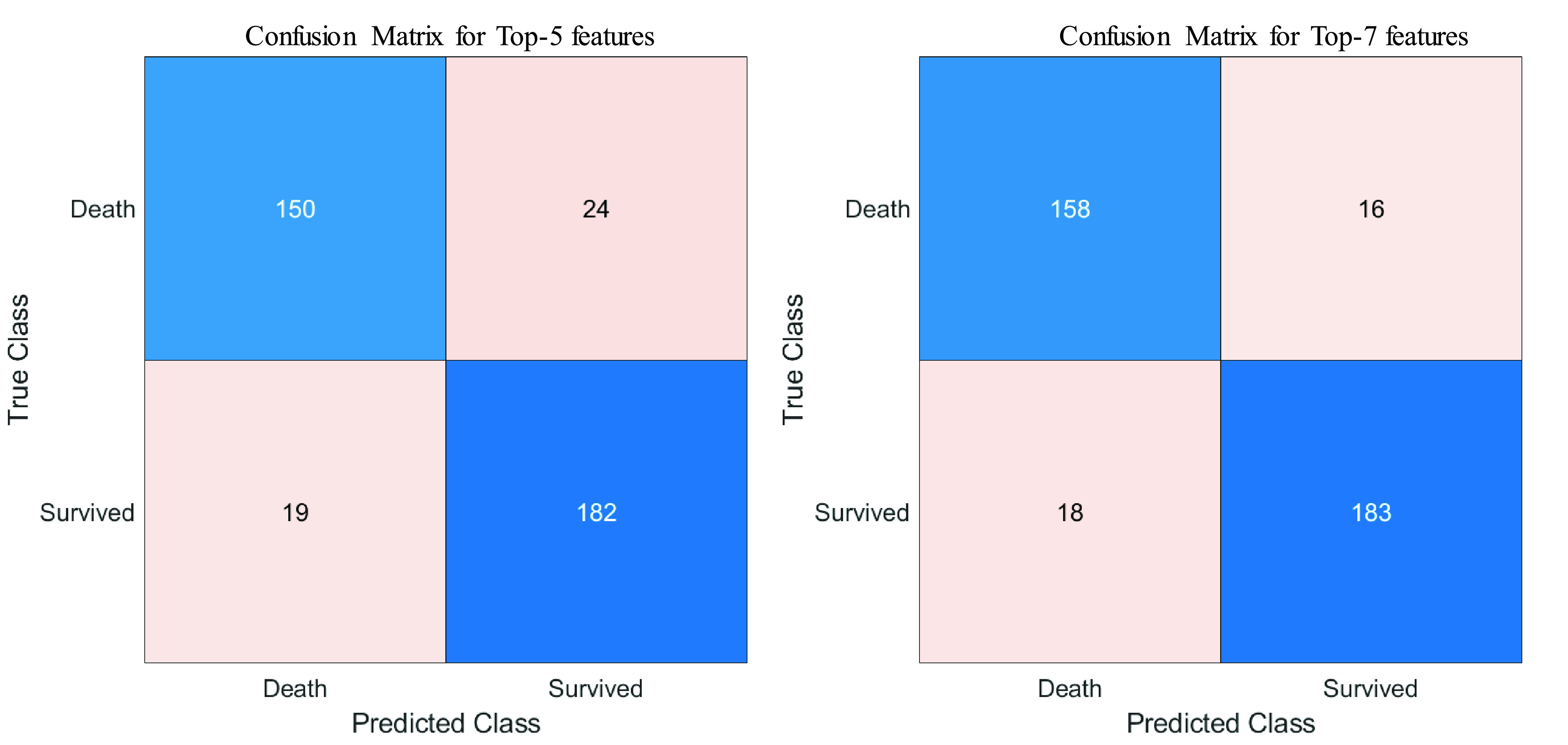
Confusion Matrix of the best performing combination of features using logistic regression classifier: A) without neutrophils and lymphocyte counts and B) without neutrophils and lymphocytes percentage.

It is clear from Figure 4B that Top-7 features without neutrophils and lymphocyte percentages while considering their count parameters provides the same AUC (0.95) as was obtained from Top-9 features while those parameters were present both in percentage and count (as shown in Figure 2). However, the percentage of neutrophils and lymphocytes did not outperform with neutrophils and lymphocytes count. This performance is further verified in Table 4, where the features with neutrophils and lymphocyte counts performed better using 7 features (Table 4). Top-ranked 7 features were: neutrophils count, lymphocyte count, age, monocyte (%), platelet count, red blood cell distribution width, and white blood cell count. The performance of the best combination of features for both the experiments can also be seen in the form of confusion matrix in Figure 5. These were used for the nomogram creation and scoring technique development and validation.

**TABLE 4:** Comparison of the Average Performance Matrix and Confusion Matrix From Five-Fold Cross-Validation for Top1 to 8 Features (A) Without Neutrophils and Lymphocyte Counts and (B) Without Neutrophils and Lymphocytes Percentage

(A)									
	Weighted Average (95% confidence interval)				Overall Accuracy	Confusion Matrix			
	Precision	Sensitivity	f1-score	Specificity		Survived		Death	
						TN	FP	FN	TP
Top 1 feature	82±4.10	84±5.18	83±6.13	84±7.08	84	168	33	27	147
Top 2 features	84±5.12	87±5.06	85±6.06	85±7.11	86	171	30	24	150
Top 3 features	88±4.12	85±6.08	86±4.05	90±5.14	87.2	181	20	28	146
Top 4 features	91.12±2.84	87±3.92	89.2±2.17	87.4±3.012	88	181	20	26	148
Top 5 features	89.2±2.03	88.04±3.13	89.2±3.03	88±3.5	88.5	182	19	24	150
Top 6 features	89±5.18	83±4.08	88±3.03	89±2.21	86.13	180	21	31	143
Top 7 features	88±4.12	85±3.08	86±4.05	90±3.14	87.2	181	20	28	146
Top 8 features	88±4.12	83±3.06	85±3.06	89±2.11	87	181	20	29	145

(B)									
	Weighted Average (95% confidence interval)				Overall Accuracy	Confusion Matrix			
	Precision	Sensitivity	f1-score	Specificity		Survived		Death	
						TN	FP	FN	TP
Top 1 feature	86±3.10	78±4.18	78±4.13	90±3.08	82	181	20	49	125
Top 2 features	88±2.12	83±3.06	85±3.06	90±2.11	87	181	20	29	145
Top 3 features	87±4.13	82±4.06	84±4.06	90±2.08	86.7	180	21	30	144
Top 4 features	88±4.12	85±2.08	86±1.05	90±4.14	87.2	181	20	28	146
Top 5 features	88±3.10	85±3.09	87±2.07	90±2.10	88	181	20	26	148
Top 6 features	90±4.18	86±3.08	88±4.03	91±2.07	89.01	183	18	24	150
Top 7 features	90±3.09	91±2.14	90±3.10	91±3.13	91	183	18	16	158
Top 8 features	88±4.15	87±3.08	86±2.14	90±2.12	88.5	181	20	23	151

### C. Logistic Regression Based Nomogram

Stata/MPv13.0 was used to derive a multivariate logistic regression-based nomogram using (1000 times) bootstrapping technique. Logistic regression coefficients, standard error, the ratio of regression coefficient and its standard error, the significance of z, 95% confidence interval (CI) of z were reported in Table 5. Z-value, which is the regression coefficient/standard error, generally shows the strength of predictors in the prediction of outcome. A high positive or negative z-value represents a strong predictor while zero represents a weak predictor. Table 5 shows that out of the 7 parameters white blood cell count is a very weak predictor while the other six variables are good predictors. However, logistic regression classifier’ performance showed in Figure 4 and Table 4 demonstrated that 7-variables outperform 5 variables. Therefore, no variable was discarded out of these 7-variables in developing the nomogram.

**TABLE 5:** The Logistic Regression Analysis to Construct the Nomogram for Death Prediction

Outcome	Coef.	Bootstrap Std. Err.	z	P> $|\text{z}|$	[95% conf. Interval]	
Neutrophils Count	.5784669	.2016622	2.87	0.004	−.1832162	.9737177
Age	.0724752	.0189537	3.82	0.000	.0353265	.1096238
Platelet Count	−.009611	.0032339	−2.97	0.003	−.0159492	−.0032728
Monocytes (%)	.0931182	.0594187	1.57	0.117	−.0233403	.2095766
White Blood Cell Count	.0064276	.1536487	0.04	0.967	−.2947183	.3075734
Lymphocyte Count	−3.567051	.912109	−3.91	0.000	−5.354752	−1.77935
Red Blood Cell Distribution Width	.7140086	.2506219	2.85	0.004	.2227986	1.205219
cons	−12.75911	3.716655	−3.43	0.001	−20.04362	−5.474599

**FIGURE 6.**
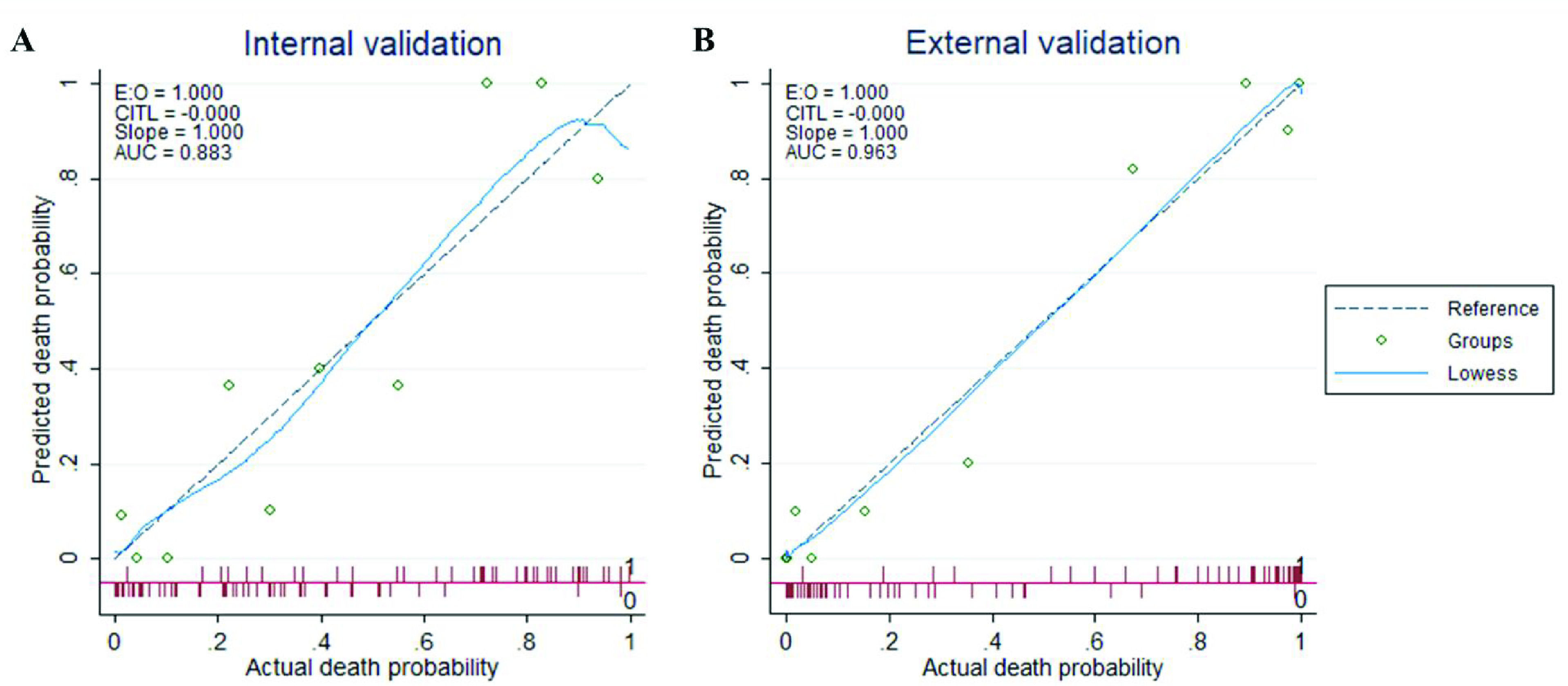
Calibration plot comparing predicted and actual death probability of patients with COVID-19: (A) represents the internal validation using Chinese test data, and (B) represents the external validation using Bangladeshi data.

**FIGURE 7.**
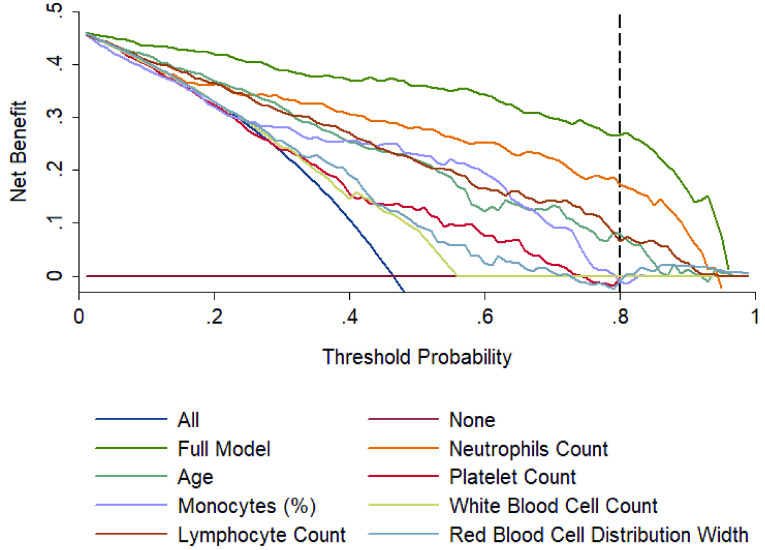
Decision curves analysis comparing different models to predict the death probability of patients with COVID-19. The net benefit balances the mortality risk and potential harm from unnecessary over-intervention for patients with COVID-19.

Figure 6 shows the calibration curves for internal (A) and external validation (B). External validation was done using the dataset collected at Dhaka Medical College and confirms the reliability of the developed model with an AUC of 0.963. The Decision Curve Analysis (DCA) can be seen in Figure 7, which can prove the clinical utility of the model. It is evident from Figure 5 that the performance is the best using the model compared to the performance using all the features or the individual features. This indicates that all of them contributed to the prediction of outcomes and also confirmed the need to combine seven predictors in the model.

**FIGURE 8.**
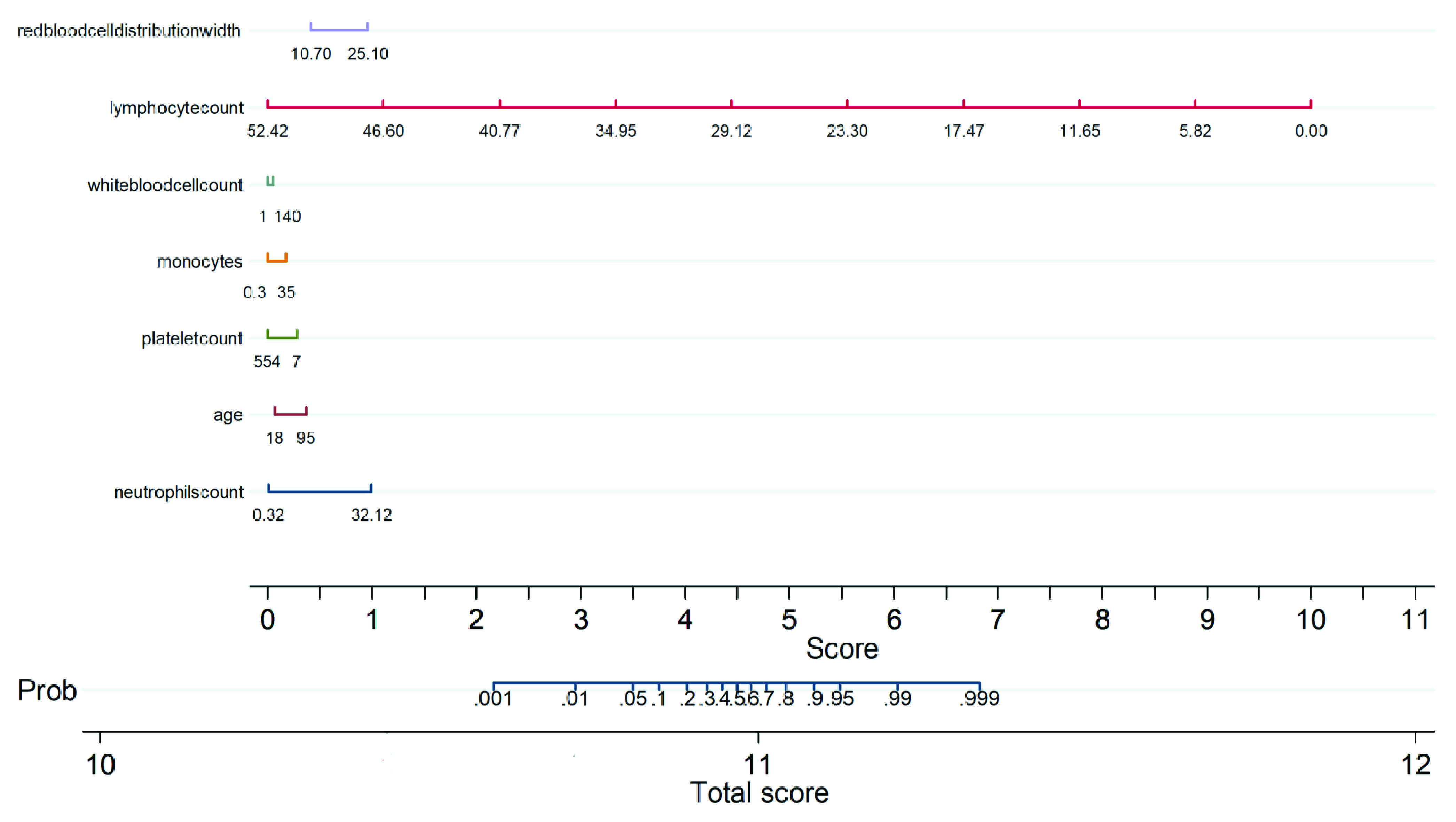
Multivariate logistic regression-based Nomogram to predict the probability of death. A Nomogram for prediction of death was created using the following seven predictors: Neutrophils count, Age, Platelet count, Monocytes, WBC, Lymphocyte count, Red blood cell distribution width.

As shown in Figure 8, the nomogram is comprised of 8 rows while rows 1–7 are representing independent variables. For each variable, an assigned score was obtained by drawing a downward vertical line from the value on the variable axis to the “Score” axis using COVID-19 patient data. The points of the seven variables correspond to the score (row 8) and the scores were added up to the total score, as shown in row 8. Then a line could be drawn from the “Total Score” axis to the “Prob” axis (row 9) to determine the death probability of COVID-19 patients. However, it is useful to derive the mathematical equations explaining the total score, linear prediction, and death probability based on which the score is calculated:

\begin{align*}&\hspace {-1.2pc}\mathbf {Linear prediction} \\=&-12.75911+0.5784669\times \text{Neutrophils Count} \\&+\,0.0724752\times \text{Age}-0.009611\times \text{Platelet Count} \\&+\,0.0931182\times \text{Monocytes}\left ({\% }\right)+0.0064276 \\&\times \text{White Blood Cell Count}-3.567051 \\&\times \text{Lymphocyte Count}+0.7140086 \\&\times \text{Red Blood Cell Distribution Width}\tag{8}\\&\hspace {-1.2pc}\mathbf {Death probability} \\=&1/ (1+\text{exp} (-\text{Linear Prediction}))\tag{9}\end{align*}

The corresponding probability of death for a given risk score was determined from the model and is listed in Table 6. In particular, risk score cut-off values of 10.8 and 10.96 corresponded to 5% and 50% of death probability, thus these values can be used to stratify COVID-19 patients into three groups: low, moderate, and high-risk groups. The death probability was less than 5%, between 5% and 50 %, and more than 50 % for the low-risk group (Score < 10.8), moderate risk group (10.8≤Score≤10.96), and high-risk group (Score > 10.96), respectively.

**TABLE 6:** The Mortality Risk Prediction Score From Nomogram and Corresponding Death Probability of COVID-19 Patients

Score	10.6	10.7	10.8	10.84	10.87	10.9	10.93	10.96	10.98	11.005	11.03	11.08	11.1	11.2	11.35
Death Probability	0.001	0.01	0.05	0.1	0.2	0.3	0.4	0.5	0.6	0.7	0.8	0.9	0.95	0.99	0.999
Risk Group	Low			Moderate					High						

### D. Performance Evaluation of the Model

**FIGURE 9.**
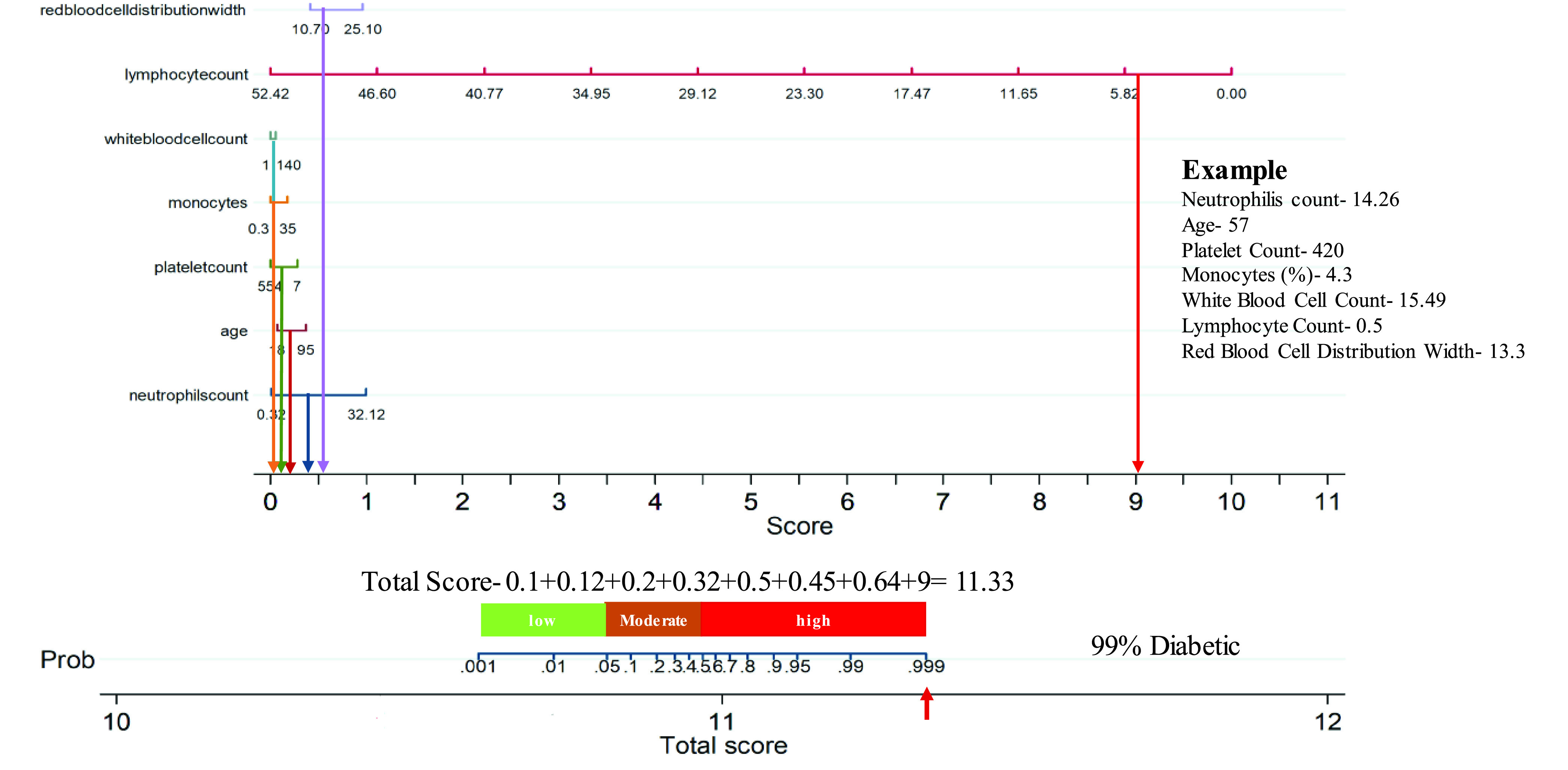
Example of the developed nomogram helping early severity classification of mortality of a patient from the dataset collected at Dhaka Medical College.

The authors have categorized, the patients from the internal cohort in training and testing subgroups as well as an external cohort into three subgroups (low, moderate, and high-risk) by associating actual outcome with the predicted outcome using the score. For the internal training set (Table 7A), the proportions of death were 1.2% (1/183) for the low-risk group, 23.33% (14/60) for the moderate-risk group, and 90.75% (108/119) for the high-risk group while for the internal test set (Table 7B), the proportions of death were 0% (0/36) for low-risk group, 21.74% (5/23) for moderate-risk group and 85.19% (46/54) for the high-risk group. For the external test set (Table 7C), the proportions of death were 0% (0/42) for the low-risk group, 26.32% (5/19) for the moderate-risk group, and 88.1% (37/42) for the high-risk group. It was found that the true death rates were significantly different (p < 0.001) among the three subgroups. Therefore, this nomogram-based scoring technique can be used to early predict patient’ outcomes to categorize them into low, moderate, and high-risk groups as shown in Table 6, and prioritize the moderate and high-risk group patients. Figure 9 shows an example nomogram-based scoring system for a COVID-19 patient with the variable values at admission. Individual scores for each predictor were calculated and added to produce the total score and death probability was calculated to 99%. This was done as early as 5 days before the death of the patient.

**TABLE 7:** Association Between Different Risk Groups and Actual Outcome in (A) The Training Cohort, (B) The Testing Cohort, and (C) The External Validation Cohort Using Fisher Exact Probability Test

(A)			
Risk category	Outcome		Overall
	Alive	Death	
Low-risk	82 (98.80%)	1 (1.2%)	83 (100.0%)
Moderate-risk	46(76.67%)	14(23.33%)	60 (100.0%)
High-risk	11 (9.25%)	108 (90.75%)	119 (100.0%)
Overall	139(53%)	123 (47%)	262 (100.0%)
P-value among the three groups is less than 0.001			
The P-value of the Low-risk group vs moderate-risk group is less than 0.001.			
The P-value of the Low-risk group vs the High-risk group is less than 0.001.			
The P-value of the Moderate-risk group vs the High-risk group is less than 0.001.			

(B)			
Risk category	Outcome		Overall
	Alive	Death	
Low-risk	36 (100%)	0 (0%)	36 (100.0%)
Moderate-risk	18 (78.26%)	5 (21.74%)	23 (100.0%)
High-risk	8 (14.81%)	46 (85.19%)	54 (100.0%)
Overall	62 (54.87%)	51 (45.13%)	113 (100.0%)
P-value among the three groups is less than 0.001			
The P-value of the Low-risk group vs moderate-risk group is less than 0.001.			
The P-value of the Low-risk group vs the High-risk group is less than 0.001.			
The P-value of the Moderate-risk group vs the High-risk group is less than 0.001.			

(C)			
Risk category	Outcome		Overall
	Alive	Death	
Low-risk	42 (100%)	0 (0%)	42 (100.0%)
Moderate-risk	14 (73.68%)	5 (26.32%)	19 (100.0%)
High-risk	5 (11.90%)	37 (88.10%)	42 (100.0%)
Overall	61 (59.22%)	42 (40.78%)	103 (100.0%)
P-value among the three groups is less than 0.001			
The P-value of the Low-risk group vs moderate-risk group is less than 0.001.			
The P-value of the Low-risk group vs the High-risk group is less than 0.001.			
The P-value of the Moderate-risk group vs the High-risk group is less than

## IV. Discussion

The current study looked into the correlation between disease severity and clinical data from complete blood count (CBC) test. Based on the data collected at the time of hospital admission, the Random Forest algorithm classified ten predictors as death probability predictors. To find the best performing classification model, these top-ranked 10 features were investigated with 6 different classifiers. Classification using logistic regression with Top-1 to Top-10 features was used to evaluate the independent variables’ relationship with the outcomes. Figure 2 clearly illustrates that the Top-9 features deliver the highest AUC (0.95) value. Table 2 demonstrates the overall accuracies and weighted average results for other matrices and the confusion matrices for various models using the Top 1 to 10 features for 5-fold cross-validation features using the logistic regression classifier. The Top-9 features-based model shows the best result with an AUC of 0.95 and overall accuracy, weighted precision, sensitivity, specificity, and F1-scores of 0.9, 0.9, 0.9, 0.91, and 0.90, respectively. Neutrophils and lymphocytes, on the other hand, were both presents in those Top-9 features in terms of percentage and count. As a result, it is important to investigate which of those features with and without the percentage of neutrophils and lymphocytes, should be used in the model development.

Previous research on the Coronavirus family, such as SARS [42], Middle East respiratory syndrome (MERS) [43], and COVID-19 [44], showed that age is a primary predictor of mortality. This research came to similar conclusions since as people get older, immunosenescence and/or multiple medical problems make them more susceptible to severe COVID-19 illness [14].

Both neutrophils and lymphocytes are essential components of the immune system since they aid in host defense and dis-infection. They can be represented in terms of count or percentage or ratio (Neutrophil-Lymphocyte Ratio –NLR). Lymphopenia, a medical disorder characterized by a reduction in the number of lymphocytes in the blood, is a common symptom in COVID-19 patients and may be a major factor in disease severity and mortality [45]. In our investigation, we have found that the percentage of neutrophils and lymphocytes were impactful and also confirmed the previous research results that a lower percentage of these two concentrations was correlated with severe COVID-19 patients [46]. Patients with community-acquired pneumonia have substantial immune system activation and/or immune dysfunction, leading to changes in these amounts [45]. Furthermore, as particular anti-inflammatory cytokines induce immunosuppression and lymphocyte apoptosis, bone marrow circulates neutrophils, resulting in a rise in NLR [47]. However, in comparison to other models, both parameters for high-risk patients were found to be small in this sample.

Lu *et al.* [6] showed to predict confirmed or suspected short-term patients mortality associated with COVID-19. Hepatocytes produce CRP in response to leukocyte-derived cytokines induced by infection, inflammation, or tissue damage [48]–[50]. It was found in this report, which assessed increased CRP levels at admission for COVID-19 patients with high mortality risk. This suggested that these patients had developed a severe lung inflammation or probably a secondary bacterial infection, which needs clinical antibiotic treatment [50].

Non-survivors had lower lymphocyte and neutrophil percentages, as well as higher age than survivors [14]. COVID-19 severity was significantly related to the inflammatory response to the infection, in addition to dysregulation of the coagulation system and/or immune system. This could result in more serious medical issues such as ARDS, septic shock, and coagulopathy among other diseases. As a result, this type of prognostic model will help in the creation of a fair and customized treatment plan for critically ill patients.

In this study, seven key predictors acquired at admission were chosen using Random forest feature selection to construct a nomogram-based prognostic model with excellent calibration and discrimination in predicting COVID-19 patients’ death probability. It was also tested on an external validation cohort. Furthermore, the model was validated using various blood sample data obtained from patients during their hospital stay, and it was found to be accurate in those cases as well. The AUC values for the development set, internal validation, and external validation cohorts were 0.954, 0.883, and 0.96, respectively. Furthermore, this nomogram-derived risk score provided a clear, easy-to-understand, and interpretable early warning method for stratifying high-risk COVID-19 patients at admission and assisting clinical management. Using this risk score assessed and determined at admission, COVID-19 patients were divided into three risk categories, each with a different risk of death. Low-risk cases could be separated and handled in an isolation unit, while moderate-risk patients could be treated in the hospital’s isolation ward. Patients in the high-risk group, on the other hand, can be closely monitored and, if possible, referred to essential medical facilities or the intensive care unit (ICU) for immediate care.

The study suggests that research on COVID-19 clinical data may aid in early mortality prediction. In this study, we have developed the model and confirmed its performance using five-fold validation. Furthermore, the model was verified with a completely unseen data from a different county and the performance was still very reliable, as can be seen from the results in Figure 6 B.

## V. Conclusion

**FIGURE 10.**
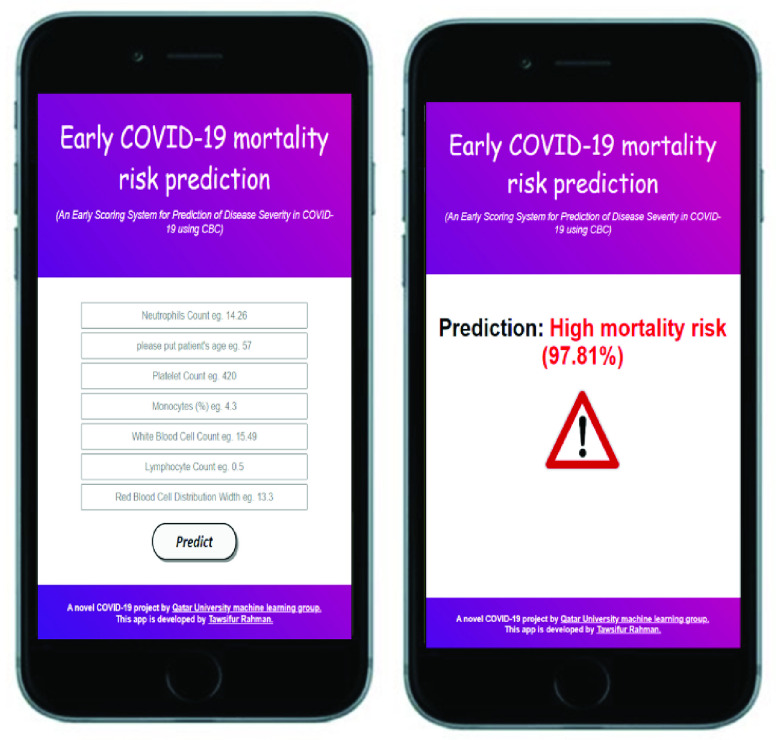
Mortality risk prediction Web-Application [51].

In conclusion, the nomogram-based scoring technique can predict the risk of COVID-19 patients with good discrimination and calibration based on multiple CBC predictors (Neutrophils count, Age, Platelet count, Monocytes, WBC, Lymphocyte count, Red blood cell distribution width). The model has a high degree of precision in predicting the patient’s outcome much earlier than the real clinical outcome. The model was tested on a completely unknown external dataset, i.e. the dataset collected from Dhaka Medical College, Bangladesh while developed on Chinese dataset. The authors have explored the various combination of feature selection technique, features and machine learning classifiers in this study with state of the art performance which was deployed as a web-application for clinical use (Figure 10). A mobile application or web-application deployment is suitable for clinical parameters compared to deep learning approach on a smaller dataset [52]. The proposed scoring technique would assist clinicians in creating an effective and optimized patient stratification management strategy without overburdening healthcare resources, as well as minimizing mortality by providing support to the severe patients earlier. The authors are collecting a multi-country and multi-center larger dataset to increase the model performance and robustness by using a large dataset.

## Disclosure of Potential Conflicts of Interest

The authors declare that they have no conflict of interest.

## Ethical Approval

This study was carried out by two clinical datasets where the development and internal validation were carried out by the publicly available dataset by Yan *et al.* [19] and external validation was done by COVID-19 clinical data collected from Dhaka Medical College hospital, Bangladesh with the ethical approval from the hospital ethics committee. COVID-19 patients’ identification was removed before the data were shared with the researchers in Qatar and medical doctors in Bangladesh were involved in data acquisition and de-identification. COVID-19 patient data collected from Wuhan Hospital by Yan *et al.* [19] was approved by the Tongji Hospital Ethics Committee.

## References

[1] (2021). Coronavirus Cases. [Online]. Available: https://www.worldometers.info/coronavirus/

[2] B. L. Krit, V. V. Kuvshinov, D. Y. Kukushkin, N. B. Morozova, Y. A. Omel’chuk, T. V. Revenok, and V. V. Sleptsov, “The application of nanoclaster coatngs for modification of image receiving surface of thermophotoelectric energy converters,” Surf. Eng. Appl. Electrochem., vol. 56, no. 1, pp. 100–104, Jan. 2020.

[3] N. Zhu, D. Zhang, W. Wang, X. Li, B. Yang, J. Song, and X. Zhao, “A novel coronavirus from patients with pneumonia in China, 2019,” New England J. Med., to be published. 10.1056/NEJMoa2001017 PMC7092803 31978945

[4] C. Huang, Y. Wang, X. Li, L. Ren, J. Zhao, Y. Hu, L. Zhang, G. Fan, J. Xu, X. Gu, Z. Cheng, T. Yu, J. Xia, Y. Wei, W. Wu, X. Xie, W. Yin, and H. Li, “Clinical features of patients infected with 2019 novel coronavirus in Wuhan, China,” Lancet, vol. 395, no. 10223, pp. 497–506, May 2020. 31986264 10.1016/S0140-6736(20)30183-5 PMC7159299

[5] W. Guan, Z.-Y. Ni, Y. Hu, W.-H. Liang, C.-Q. Ou, J.-X. He, L. Liu, H. Shan, C.-L. Lei, D. S. C. Hui, B. Du, and L.-J. Li, “Clinical characteristics of coronavirus disease 2019 in China,” New England J. Med., vol. 382, no. 18, pp. 1708–1720, 2020. 32109013 10.1056/NEJMoa2002032 PMC7092819

[6] J. Lu, S. Hu, R. Fan, Z. Liu, X. Yin, Q. Wang, Q. Lv, Z. Cai, H. Li, Y. Hu, and Y. Han, “ACP risk grade: A simple mortality index for patients with confirmed or suspected severe acute respiratory syndrome coronavirus 2 disease (COVID-19) during the early stage of outbreak in Wuhan, China,” Tech. Rep., 2020.

[7] N. Chen, M. Zhou, X. Dong, J. Qu, F. Gong, Y. Han, and Y. Qiu, “Epidemiological and clinical characteristics of 99 cases of 2019 novel coronavirus pneumonia in Wuhan, China: A descriptive study,” Lancet, vol. 395, no. 10223, pp. 507–513, May 2020. 32007143 10.1016/S0140-6736(20)30211-7 PMC7135076

[8] J. F. W. Chan, S. Yuan, K.-H. Kok, K. W. Kelvin, H. Chu, J. Yang, F. Xing, J. Liu, C. Chik-Yan, R. Wing-Shan, W.-M. Chan, and J. Daniel, “A familial cluster of pneumonia associated with the 2019 novel coronavirus indicating person-to-person transmission: A study of a family cluster,” Lancet, vol. 395, no. 10223, pp. 514–523, Feb. 2020. 31986261 10.1016/S0140-6736(20)30154-9 PMC7159286

[9] D. Wang, B. Hu, C. Hu, F. Zhu, X. Liu, J. Zhang, and B. Wang, “Clinical characteristics of 138 hospitalized patients with 2019 novel coronavirus–infected pneumonia in Wuhan, China,” JAMA, vol. 323, no. 11, pp. 1061–1069, 2020. 32031570 10.1001/jama.2020.1585 PMC7042881

[10] A. Iasonos, D. Schrag, G. V. Raj, and K. S. Panageas, “How to build and interpret a nomogram for cancer prognosis,” J. Clin. Oncol., vol. 26, no. 8, pp. 1364–1370, Mar. 2008. 18323559 10.1200/JCO.2007.12.9791

[11] J. Wu, J. Qiu, W. Jiang, J. Qiu, L. Zhang, R. Zhao, and C. Yu, “Development and validation of a nomogram predicting the probability of type a aortic dissection at a diameter below 55 mm: A retrospective cohort study,” Int. J. Surgery, vol. 60, pp. 266–272, Dec. 2018. 10.1016/j.ijsu.2018.11.024 30496867

[12] Y. Zheng, Y. Zhang, H. Chi, S. Chen, M. Peng, L. Luo, L. Chen, J. Li, B. Shen, and D. Wang, “The hemocyte counts as a potential biomarker for predicting disease progression in COVID-19: A retrospective study,” Clin. Chem. Lab. Med. (CCLM), vol. 58, no. 7, pp. 1106–1115, Jun. 2020. 32352397 10.1515/cclm-2020-0377

[13] S. Al Youha, S. A. Doi, M. H. Jamal, S. Almazeedi, M. Al Haddad, M. AlSeaidan, A. Y. Al-Muhaini, F. Al-Ghimlas, and S. K. Al-Sabah, “Validation of the Kuwait progression indicator score for predicting progression of severity in COVID19,” medRxiv, to be published.

[14] Z. Weng, Q. Chen, S. Li, H. Li, Q. Zhang, S. Lu, L. Wu, L. Xiong, B. Mi, D. Liu, M. Lu, D. Yang, H. Jiang, S. Zheng, and X. Zheng, “ANDC: An early warning score to predict mortality risk for patients with coronavirus disease 2019,” J. Transl. Med., vol. 18, no. 1, pp. 1–10, Dec. 2020. 32867787 10.1186/s12967-020-02505-7 PMC7457219

[15] P. Ramachandran, M. Gajendran, A. Perisetti, K. O. Elkholy, A. Chakraborti, G. Lippi, and H. Goyal, “Red blood cell distribution width (RDW) in hospitalized COVID-19 patients,” medRxiv, to be published. 10.3389/fmed.2021.582403 PMC8778579 35071250

[16] J. Gong, J. Ou, X. Qiu, Y. Jie, Y. Chen, L. Yuan, J. Cao, M. Tan, W. Xu, F. Zheng, Y. Shi, and B. Hu, “A tool for early prediction of severe coronavirus disease 2019 (COVID-19): A multicenter study using the risk nomogram in Wuhan and Guangdong, China,” Clin. Infectious Diseases, vol. 71, no. 15, pp. 833–840, Jul. 2020. 10.1093/cid/ciaa443 PMC7184338 32296824

[17] B. H. Foy, J. C. T. Carlson, E. Reinertsen, R. P. Valls, E. Palanques-Tost, C. Mow, M. B. Westover, A. D. Aguirre, and J. M. Higgins, “Elevated RDW is associated with increased mortality risk in COVID-19,” medRxiv, to be published.

[18] X. Jianfeng, D. Hungerford, H. Chen, S. T. Abrams, S. Li, and G. Wang, “Development and external validation of a prognostic multivariable model on admission for hospitalized patients with COVID-19,” medRxiv. to be published. [Online]. Available: https://www.medrxiv.org/content/medrxiv/early/2020/03/30/2020.03.28.20045997.full.pdf

[19] L. Yan, H. T. Zhang, J. Goncalves, Y. Xiao, M. Wang, Y. Guo, C. Sun, X. Tang, L. Jing, M. Zhang, and X. Huang, “An interpretable mortality prediction model for COVID-19 patients,” Nature Mach. Intell., vol. 2, no. 5, pp. 283–288, May 2020.

[20] W. Liang, J. Yao, and J. He, “Early triage of critically ill COVID-19 patients using deep learning,” Nature Commun., vol. 11, no. 1, p. 3543, 2020. 32669540 10.1038/s41467-020-17280-8 PMC7363899

[21] C. Wang, R. Deng, L. Gou, Z. Fu, X. Zhang, F. Shao, G. Wang, W. Fu, J. Xiao, X. Ding, T. Li, X. Xiao, and C. Li, “Preliminary study to identify severe from moderate cases of COVID-19 using combined hematology parameters,” Ann. Transl. Med., vol. 8, no. 9, p. 593, May 2020. 32566620 10.21037/atm-20-3391 PMC7290538

[22] D. Huang, T. Wang, Z. Chen, H. Yang, R. Yao, and Z. Liang, “A novel risk score to predict diagnosis with coronavirus disease 2019 (COVID-19) in suspected patients: A retrospective, multicenter, and observational study,” J. Med. Virol., vol. 92, no. 11, pp. 2709–2717, 2020. 32510164 10.1002/jmv.26143 PMC7300577

[23] Y.-Q. Cai, H.-Q. Zeng, X.-B. Zhang, X.-J. Wei, L. Hu, Z.-Y. Zhang, Q. Ming, Q.-P. Peng, and L.-D. Chen, “Prognostic value of neutrophil-to-lymphocyte ratio, lactate dehydrogenase, D-dimer and CT score in patients with COVID-19,” Tech. Rep., 2020. 10.18632/aging.203501 PMC8457612 34495869

[24] Y.-P. Liu, G.-M. Li, J. He, Y. Liu, M. Li, R. Zhang, Y.-L. Li, Y.-Z. Wu, and B. Diao, “Combined use of the neutrophil-to-lymphocyte ratio and CRP to predict 7-day disease severity in 84 hospitalized patients with COVID-19 pneumonia: A retrospective cohort study,” Ann. Translational Med., vol. 8, no. 10, p. 635, May 2020. 10.21037/atm-20-2372 PMC7290615 32566572

[25] C. Zhang, L. Qin, K. Li, Q. Wang, Y. Zhao, B. Xu, L. Liang, Y. Dai, Y. Feng, J. Sun, X. Li, Z. Hu, H. Xiang, T. Dong, R. Jin, and Y. Zhang, “A novel scoring system for prediction of disease severity in COVID-19,” Frontiers Cellular Infection Microbiol., vol. 10, p. 318, Jun. 2020. 10.3389/fcimb.2020.00318 PMC7292148 32582575

[26] Y. Shang, T. Liu, Y. Wei, J. Li, L. Shao, M. Liu, Y. Zhang, Z. Zhao, H. Xu, Z. Peng, X. Wang, and F. Zhou, “Scoring systems for predicting mortality for severe patients with COVID-19,” EClinicalMedicine, vol. 24, Jul. 2020, Art. no. 100426. 10.1016/j.eclinm.2020.100426 PMC7332889 32766541

[27] M. P. McRae, G. W. Simmons, N. J. Christodoulides, Z. Lu, S. K. Kang, D. Fenyo, T. Alcorn, I. P. Dapkins, I. Sharif, D. Vurmaz, S. S. Modak, K. Srinivasan, S. Warhadpande, R. Shrivastav, and J. T. McDevitt, “Clinical decision support tool and rapid point-of-care platform for determining disease severity in patients with COVID-19,” Lab a Chip, vol. 20, no. 12, pp. 2075–2085, Jun. 2020. 10.1039/d0lc00373e PMC7360344 32490853

[28] L. Zhang, X. Yan, Q. Fan, H. Liu, X. Liu, Z. Liu, and Z. Zhang, “D-dimer levels on admission to predict in-hospital mortality in patients with COVID-19,” J. Thrombosis Haemostasis, vol. 18, no. 6, pp. 1324–1329, 2020. 10.1111/jth.14859 PMC7264730 32306492

[29] (2021). COVID-19 Complete Blood Count (CBC) Database. Accessed: Jul. 9, 2021. [Online]. Available: https://www.kaggle.com/tawsifurrahman/covid19-complete-blood-count-clinical-database

[30] H. Hegde, N. Shimpi, A. Panny, I. Glurich, P. Christie, and A. Acharya, “MICE vs PPCA: Missing data imputation in healthcare,” Informat. Med. Unlocked, vol. 17, Jan. 2019, Art. no. 100275. 10.1016/j.imu.2019.100254 PMC7453822 32864420

[31] J. R. Stevens, A. Suyundikov, and M. L. Slattery, “Accounting for missing data in clinical research,” Jama, vol. 315, no. 5, pp. 517–518, 2016. 26836740 10.1001/jama.2015.16461 PMC6010186

[32] J. L. Speiser, M. E. Miller, J. Tooze, and E. Ip, “A comparison of random forest variable selection methods for classification prediction modeling,” Expert Syst. Appl., vol. 134, pp. 93–101, Nov. 2019. 32968335 10.1016/j.eswa.2019.05.028 PMC7508310

[33] Q. Gu, Z. Li, and J. Han, “Linear discriminant dimensionality reduction,” in Proc. Joint Eur. Conf. Mach. Learn. Knowl. Discovery Databases, 2011, pp. 549–564.

[34] L. Breiman, “Random forests,” Mach. Learn., vol. 45, no. 1, pp. 5–32, 2001.

[35] Y. Zhang, “Support vector machine classification algorithm and its application,” in Proc. Int. Conf. Inf. Comput. Appl., 2012, pp. 179–186.

[36] X. Shi, Q. Li, Y. Qi, T. Huang, and J. Li, “An accident prediction approach based on XGBoost,” in Proc. 12th Int. Conf. Intell. Syst. Knowl. Eng. (ISKE), Nov. 2017, pp. 1–7.

[37] C. Subasi. Logistic Regression Classifier. Accessed: Jun. 1, 2021. [Online]. Available: https://towardsdatascience.com/logistic-regression-classifier-8583e0c3cf9

[38] S. K. Pal and S. Mitra, “Multilayer perceptron, fuzzy sets, and classification,” IEEE Trans. Neural Netw., vol. 3, no. 5, pp. 683–697, 1992, doi: 10.1109/72.159058. 18276468

[39] N. V. Chawla, K. W. Bowyer, L. O. Hall, and W. P. Kegelmeyer, “SMOTE: Synthetic minority over-sampling technique,” J. Artif. Intell. Res., vol. 16, no. 1, pp. 321–357, 2002.

[40] Q. Zou, S. Xie, Z. Lin, M. Wu, and Y. Ju, “Finding the best classification threshold in imbalanced classification,” Big Data Res., vol. 5, pp. 2–8, Sep. 2016.

[41] A. Zlotnik and V. Abraira, “A general-purpose nomogram generator for predictive logistic regression models,” Stata J.: Promoting Commun. Statist. Stata, vol. 15, no. 2, pp. 537–546, Jun. 2015.

[42] J. C. Chan, E. L. Tsui, and V. C. Wong, “Prognostication in severe acute respiratory syndrome: A retrospective time-course analysis of 1312 laboratory-confirmed patients in Hong Kong,” Respirology, vol. 12, no. 4, pp. 531–542, 2007. 17587420 10.1111/j.1440-1843.2007.01102.x PMC7192325

[43] A. Assiri, J. A. Al-Tawfiq, A. A. Al-Rabeeah, F. A. Al-Rabiah, S. Al-Hajjar, A. Al-Barrak, H. Flemban, W. N. Al-Nassir, H. H. Balkhy, R. F. Al-Hakeem, H. Q. Makhdoom, A. I. Zumla, and Z. A. Memish, “Epidemiological, demographic, and clinical characteristics of 47 cases of middle east respiratory syndrome coronavirus disease from Saudi Arabia: A descriptive study,” Lancet Infectious Diseases, vol. 13, no. 9, pp. 752–761, Sep. 2013. 23891402 10.1016/S1473-3099(13)70204-4 PMC7185445

[44] R. Chen, W. Liang, M. Jiang, W. Guan, C. Zhan, T. Wang, C. Tang, L. Sang, J. Liu, Z. Ni, Y. Hu, L. Liu, H. Shan, C. Lei, Y. Peng, L. Wei, Y. Liu, Y. Hu, and N. Zhong, “Risk factors of fatal outcome in hospitalized subjects with coronavirus disease 2019 from a nationwide analysis in China,” Chest, vol. 158, no. 1, pp. 97–105, Jul. 2020. 32304772 10.1016/j.chest.2020.04.010 PMC7158802

[45] I. Huang and R. Pranata, “Lymphopenia in severe coronavirus disease-2019 (COVID-19): Systematic review and meta-analysis,” J. Intensive Care, vol. 8, no. 1, pp. 1–10, Dec. 2020. 32483488 10.1186/s40560-020-00453-4 PMC7245646

[46] J. Liu, Y. Liu, P. Xiang, L. Pu, H. Xiong, C. Li, and M. Zhang, “Neutrophil-to-lymphocyte ratio predicts critical illness patients with 2019 coronavirus disease in the early stage,” J. Transl. Med., vol. 18, no. 1, pp. 1–12, Dec. 2020. 32434518 10.1186/s12967-020-02374-0 PMC7237880

[47] M. Adamzik, J. Broll, J. Steinmann, A. M. Westendorf, I. Rehfeld, C. Kreissig, and J. Peters, “An increased alveolar CD4+ CD25+ Foxp3+ T-regulatory cell ratio in acute respiratory distress syndrome is associated with increased 30-day mortality,” Intensive care Med., vol. 39, no. 10, pp. 1743–1751, 2013. 23949701 10.1007/s00134-013-3036-3 PMC7095258

[48] J.-H. Ko, G. E. Park, J. Y. Lee, J. Y. Lee, S. Y. Cho, Y. E. Ha, C.-I. Kang, J.-M. Kang, Y.-J. Kim, H. J. Huh, C.-S. Ki, B.-H. Jeong, J. Park, C. R. Chung, D. R. Chung, J.-H. Song, and K. R. Peck, “Predictive factors for pneumonia development and progression to respiratory failure in MERS-CoV infected patients,” J. Infection, vol. 73, no. 5, pp. 468–475, Nov. 2016. 10.1016/j.jinf.2016.08.005 PMC7112644 27519621

[49] J. Wang, X. Wu, Y. Tian, X. Li, X. Zhao, and M. Zhang, “Dynamic changes and diagnostic and prognostic significance of serum PCT, Hs-CRP and S-100 protein in central nervous system infection,” Exp. Therapeutic Med., vol. 16, no. 6, pp. 5156–5160, 2018. 10.3892/etm.2018.6866 PMC6256862 30546413

[50] B. Yildiz, H. Poyraz, N. Cetin, N. Kural, and O. Colak, “High sensitive C-reactive protein: A new marker for urinary tract infection, VUR and renal scar,” Eur. Rev. Med. Pharmacol. Sci., vol. 17, no. 19, pp. 2598–2604, 2013. 24142605

[51] T. Rahman. Early COVID-19 Mortality Risk Prediction. Accessed: Jun. 1, 2021. [Online]. Available: https://COVID-19-risk-prediction.herokuapp.com/

[52] A. Khandakar, M. E. H. Chowdhury, M. B. I. Reaz, S. H. Ali, M. A. Hasan, S. Kiranyaz, T. Rahman, R. Alfkey, A. A. A. Bakar, and R. A. Malik, “A machine learning model for early detection of diabetic foot using thermogram images,” 2021, arXiv:2106.14207. [Online]. Available: http://arxiv.org/abs/2106.14207 10.1016/j.compbiomed.2021.104838 34534794

